# Improving Device Characteristics of Dual-Gate IGZO Thin-Film Transistors with Ar–O_2_ Mixed Plasma Treatment and Rapid Thermal Annealing

**DOI:** 10.3390/membranes12010049

**Published:** 2021-12-30

**Authors:** Wei-Sheng Liu, Chih-Hao Hsu, Yu Jiang, Yi-Chun Lai, Hsing-Chun Kuo

**Affiliations:** 1 Department of Electrical Engineering, Yuan Ze University, Chung-Li 320, Taiwan; dark201314@gmail.com (C.-H.H.); zgyf742647518@gmail.com (Y.J.); egin637@gmail.com (Y.-C.L.); 2 Department of Nursing, Division of Basic Medical Sciences, Chang Gung University of Science and Technology, Chiayi 613, Taiwan; 3 Chang Gung Memorial Hospital, Chiayi 613, Taiwan; 4 Research Center for Food and Cosmetic Safety, College of Human Ecology, Chang Gung University of Science and Technology, Taoyuan 333, Taiwan; 5 Chronic Diseases and Health Promotion Research Center, Chang Gung University of Science and Technology, Chiayi 613, Taiwan

**Keywords:** indium–gallium–zinc oxide (IGZO), plasma treatment, dual-gate thin-film transistor (DG TFT)

## Abstract

In this study, high-performance indium–gallium–zinc oxide thin-film transistors (IGZO TFTs) with a dual-gate (DG) structure were manufactured using plasma treatment and rapid thermal annealing (RTA). Atomic force microscopy measurements showed that the surface roughness decreased upon increasing the O_2_ ratio from 16% to 33% in the argon–oxygen plasma treatment mixture. Hall measurement results showed that both the thin-film resistivity and carrier Hall mobility of the Ar–O_2_ plasma–treated IGZO thin films increased with the reduction of the carrier concentration caused by the decrease in the oxygen vacancy density; this was also verified using X-ray photoelectron spectroscopy measurements. IGZO thin films treated with Ar–O_2_ plasma were used as channel layers for fabricating DG TFT devices. These DG IGZO TFT devices were subjected to RTA at 100 °C–300 °C for improving the device characteristics; the field-effect mobility, subthreshold swing, and I_ON_/I_OFF_ current ratio of the 33% O_2_ plasma–treated DG TFT devices improved to 58.8 cm^2^/V·s, 0.12 V/decade, and 5.46 × 10^8^, respectively. Long-term device stability reliability tests of the DG IGZO TFTs revealed that the threshold voltage was highly stable.

## 1. Introduction

Owing to rapid developments in optoelectronic technology, the latest-generation displays are tending toward having thinner, lighter, and larger screens. In this regard, thin-film transistors (TFTs) with excellent device performance have received significant attention [1,2,3,4,5,6]. Amorphous indium–gallium–zinc oxide (a-IGZO) thin films have been employed as the channel layer in the fabrication of IGZO TFT devices, and they are expected to be applied in next-generation flat panel displays (such as 8K televisions with high frame rate, large outdoor display panels, and mobile devices with flexible display panels) because of their excellent electrical characteristics such as high optical transparency, high field-effect carrier mobility, low manufacturing cost, and possibility of being manufactured at low temperature with the characteristic uniformity of large-area displays [7]. COMPARED to the typically used amorphous silicon TFTs, a-IGZO TFTs have considerably higher field-effect carrier mobility and device operational stability and can be fabricated at lower processing temperatures [8,9,10,11,12,13].

In the fabrication of IGZO TFTs, glass substrates are generally used, adopted with a sputter deposited IGZO thin film as the channel layer. However, in the continuous fabrication of IGZO TFTs, glass substrates are disadvantageous because they are fragile. Although thin-glass substrates have elasticity at thicknesses of less than 200 μm, achieving a roll-to-roll process is difficult, and the substrates crack easily during the manufacturing process. Therefore, researchers are still attempting to incorporate IGZO TFTs into flexible substrates for subsequent application in wearable devices [14,15,16,17,18,19,20,21,22,23].

The development of IGZO TFTs on plastic substrates such as polyimide, polyethylene terephthalate, and polyethylene naphthalene has progressed rapidly to the point at which flexible display panels have been recently manufactured for use in wearable devices [24]. Compared with glass substrates, plastic substrates have high transparency and favorable surface flatness; moreover, they have stable chemical properties. Plastic substrates are also easily bent, making them suitable for the manufacturing of flexible devices in a roll-to-roll process. These excellent characteristics of plastic substrates make them an attractive substitute for glass substrates [25,26,27,28].

As mentioned, plastic substrates are suitable for the manufacture of flexible IGZO TFT devices. However, a high-temperature thermal annealing process (>400 °C) is usually required to ensure that the electrical properties of TFTs are favorable. Unfortunately, the thermal expansion coefficient of plastic substrates (50 ppm/°C) is much higher than that of glass substrates (0.55 ppm/°C). Therefore, the manufacture of IGZO TFTs on plastic substrates by using high-temperature processing could lead to softening, deformation, melting, or decomposition of the plastic substrate, resulting in degraded electrical characteristics of the resulting TFT device or process failure. Therefore, reducing the process temperature during the manufacture of devices containing plastic substrates while maintaining or even enhancing the performance of TFT devices is essential.

One study performed oxygen plasma treatment of IGZO thin films to reduce the films’ density of oxygen vacancies at low process temperatures and thereby improve the quality of the thin-film materials and the performance of IGZO TFT devices [29]. According to the results from recent plasma treatment studies, hydrogen plasma can passivate interface trap density and oxygen vacancy defects of the IGZO channel, as well as increase carrier mobility. Additionally, since the ionic radius of the nitrogen atom is close to that of the O atom, the N atom can act as a defect binder and effectively reduce the oxygen vacancy density in the oxide film. An appropriate composition of N/H plasma treatment was also studied for effectively reducing the density of traps at the SiO_2_/α-IGZO interface and passivating the oxygen vacancy-related defects of α-IGZO TFTs. Nevertheless, excessive N/H plasma treatment may lead to interstitial defects at the SiO_2_/α-IGZO interface and degrade the IGZO: N/H TFT devices' electrical characteristics [30,31].

By treating the surface of IGZO TFTs with oxygen plasma, the density of oxygen vacancies can be reduced, improving the field-effect carrier mobility and current switching ratio of the TFT device. However, as the plasma treatment time increases, O^2+^ will aggregate at the surface of IGZO serving as the trapping centers and preventing subsequent atomic oxygen from filling the oxygen vacancies and degrading device characteristics. Therefore, argon gas is added to the oxygen plasma as the Ar/O_2_ mixture for removing the O^2+^ that is covered on the IGZO channel layer for further reducing the oxygen vacancy [32]. In our previous study, the IGZO thin films with amorphous structure were subjected to the Ar/O_2_ plasma mixture treatment with varied ratios of oxygen composition and demonstrating the improved IGZO bottom-gate TFT device operation characteristics [33].

Nevertheless, the IGZO thin-film surface was severely damaged; in particular, ion bombardment during the plasma treatment increased the surface roughness. This could result in degraded electric characteristics of TFTs, reduced field-effect mobility, and more leakage current paths and carrier trapping centers because of increased surface scattering effects in the plasma-treated IGZO TFTs. Therefore, modulation of the position of the carrier transportation path in the IGZO channel layer to prevent the transporting carriers in the IGZO channel layer from being affected by the scattering effect of the damaged thin-film surface is necessary and important.

Single-gate (SG) IGZO TFT is widely used for high-definition active-matrix liquid crystal displays (AMLCD) and active-matrix light emitting diodes (AMOLED) because of its high field-effect mobility (>10 cm^2^/V·s), low off-state current and low subthreshold swing. However, there are still issues related to the consistency of various properties like bias, temperature, and the performance of the device. To improve the device operation characteristics and reliability of IGZO TFT, a dual-gate (DG) design for the TFT with enhanced threshold voltage control capability as a DG TFT was fabricated and employed for further investigation [34]. Recently, DG IGZO TFTs have attracted great attention owing to their remarkable advantages, such as excellent control of low turn-on voltage (V_ON_) and enhancement of turn-on current (I_ON_; i.e., gm value), owing to the formation of the two channels formed by the simultaneously applied voltages on the upper and lower gate electrodes. Therefore, increasing the flexibility of the electrical circuit is possible, as is modulating the channel layer position by simultaneously applying the respective voltages to the upper and lower gate electrodes. This results in significant improvement of the drain current owing to the formation of parallel channel layers compared with the single-channel layers in SG IGZO TFT devices. Moreover, the influence of the interfacial carrier capture centers and the carrier surface scattering effect at the insulator–channel layer interface could be dramatically mitigated in DG TFTs by modulating the channel layer position as well as the carrier transportation path, thereby contributing to the enhancement of carrier mobility and reduction of the subthreshold swing (*S.S.*) of the TFT device [35].

In this study, a DG structure was used to modulate the position of the channel layer in an IGZO TFT to reduce the surface scattering resulting from damage to the IGZO thin-film surface caused by argon–oxygen (Ar–O_2_) mixed plasma treatment. Additionally, low-temperature rapid thermal annealing (RTA) was employed to improve further the performance characteristics of the plasma-treated DG IGZO TFT; the resultant field-effect carrier mobility was 58.8 cm^2^/V·s, *S.S.* was 0.12 V/decade, and I_ON_/I_OFF_ current ratio was 5.46 × 10^8^.

## 2. Materials and Methods

**Figure 1:**
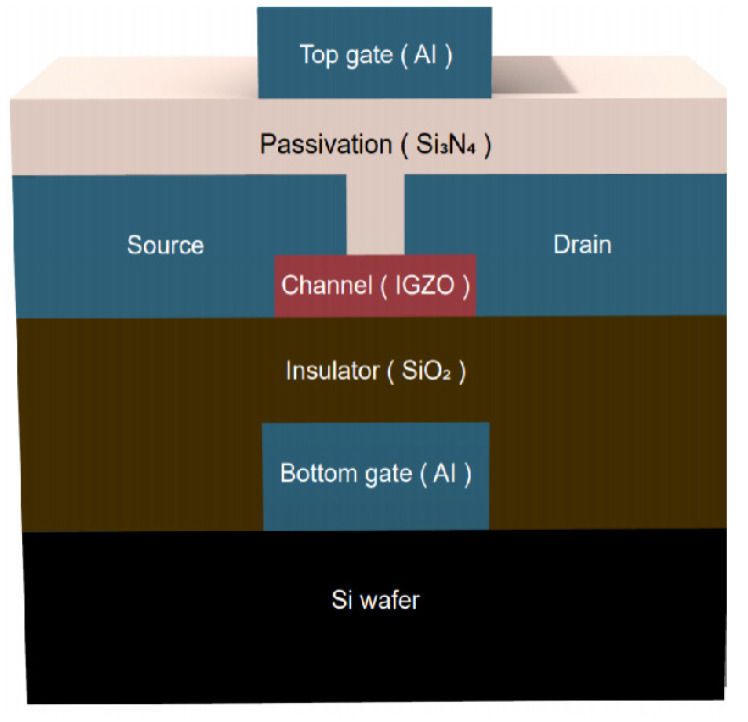
Schematic of an IGZO TFT with DG design.

The IGZO TFTs investigated in this study were fabricated to have an SG or DG structure, and the devices’ characteristics were compared in terms of the field-effect carrier mobility, *S.S.*, and I_ON_/I_OFF_ current ratio. The SG IGZO TFT was fabricated as follows. First, a 50 nm-thick aluminum gate, to act as the bottom gate electrode, was deposited using an E-gun system onto a 500 nm-thick Si wafer. Then, plasma-enhanced chemical vapor deposition (PECVD) was used to deposit a 250 nm-thick SiO_2_ layer on the bottom gate. Subsequently, 50 nm-thick IGZO channel layers were deposited using radio frequency magnetron sputtering with an In_2_Ga_2_ZnO_7_ (In_2_O_3_:Ga_2_O_3_:ZnO = 1:1:1 mol%) target at a sputtering power of 50 W for 1500 s, and the thin-film deposition working pressure was kept at 5 × 10^−3^ Torr with Ar/O_2_ (32/1 sccm) plasma. Next, the sputter-deposited IGZO thin films were subjected to Ar–O_2_ mixed plasma treatment with an O_2_ gas flow ratio of 16%, 20%, or 33% (respectively denoted as samples A, B, and C) in a high-density plasma (HDP) system with HDP set at 100 W for 20 s at the working pressure of 5 × 10^−3^ Torr; the IGZO thin film without plasma treatment was denoted the pristine sample. The surface morphology of the IGZO thin films was studied using atomic force microscopy (AFM). Next, 300 nm-thick aluminum was deposited as the source and drain electrodes on the IGZO channel layer by using the E-gun system, completing the fabrication of the SG IGZO TFT. The channel length (L) and width (W) of the IGZO TFTs were 50 and 500 μm, respectively. The DG IGZO TFT structure was fabricated in the same manner as the SG TFTs but with a 250 nm-thick Si_3_N_4_ layer deposited using PECVD and acting as the passivation layer (top-gate insulator). Finally, a 50 nm-thick aluminum layer was deposited as the top gate electrode. Figure 1 illustrates the structure of a DG IGZO TFT.

After manufacturing the TFT devices, a post thermal annealing process was performed on both the SG and DG IGZO TFT devices with annealing temperatures of 100–300 °C for 2 h under ambient nitrogen. The heating rate and cooling rate during the thermal annealing process were 15 °C/min and 10 °C/min, respectively. The chemical composition of the IGZO thin films were analyzed using X-ray photoemission spectroscopy (XPS), respectively. The B1500A semiconductor parameter analysis instrument was employed to thoroughly evaluate and compare the performance of the TFT devices. 

## 3. Results and Discussion

### 3.1. XPS

**Figure 2:**
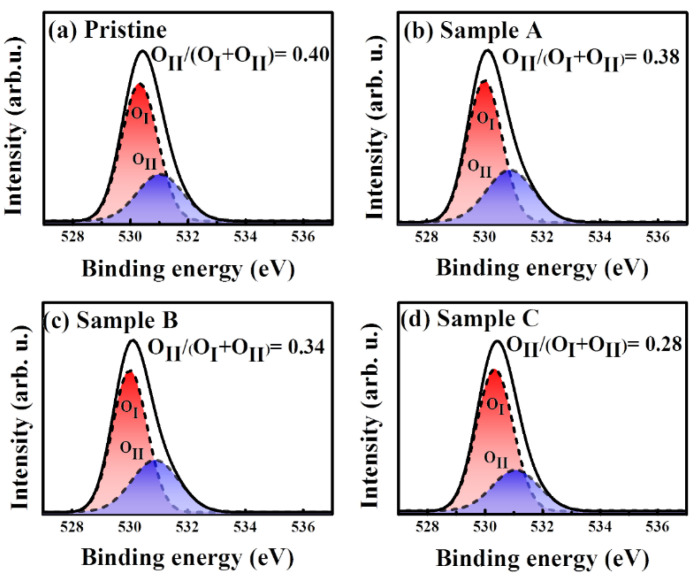
High-resolution O 1s XPS spectra with curve-fitting results obtained for IGZO thin films: (**a**) pristine sample and (**b**) sample A, (**c**) sample B, and (**d**) sample C.

XPS was employed to investigate the chemical characteristics of plasma treated IGZO thin films obtained using various oxygen flow ratios. The relationship between the oxygen flow ratio and oxygen deficiency during the plasma treatment was investigated comprehensively by comparing the high-resolution scans of the O 1s XPS line in Figure 2. Figure 2a shows the XPS spectrum of the O 1s peak of the pristine sample. The XPS O 1s peak of samples A, B, and C is shown in Figure 2b–d, respectively.

The O 1s core levels of the IGZO thin films exhibited asymmetrical high binding energy and consisted of two mixed Gaussian–Lorentzian functions, corresponding to O_I_ and O_II_, with their centers at 530.3 and 531.3 eV, respectively [36]. The O_I_ peak represents the covalent bond of oxygen ions with Zn, Ga, and In cations, whereas the O_II_ XPS signal peak represents oxygen deficiencies. Therefore, the integrated peak ratio of the O_II_/(O_I_ + O_II_) intensity ratio of the O 1s core levels of the IGZO thin films was used to evaluate thin-film quality and the number of oxygen deficiencies in the plasma-treated IGZO thin films. The calculated XPS O_II_/(O_I_ + O_II_) intensity ratio of the pristine sample was 0.40, whereas the intensity ratio was lower at 0.38, 0.34, and 0.28 for the plasma-treated IGZO thin films with increased oxygen flow ratios in samples A, B, and C, respectively.

The low O_II_/(O_I_ + O_II_) XPS intensity ratios revealed markedly fewer oxygen deficiencies and higher quality of the IGZO thin films when oxygen plasma treatment was applied at a higher oxygen flow ratio. Because oxygen vacancies play the critical role of donating electrons in a-IGZO thin films, the lower O_II_ peak intensity observed in the XPS measurements agreed well with the lower carrier concentration and higher carrier mobility for sample C in the Hall measurement. Therefore, as the oxygen content in the plasma treatment increases, the reduced carrier concentration in the IGZO film could result in the increased threshold voltage (V_TH_) of the IGZO TFT device [33,37].

The chemical stoichiometry and oxygen content should be suitable when preparing a-IGZO thin films for the fabrication of TFT devices because oxygen deficiencies are closely related to the formation of donor states and deep-level electronic traps, which strongly affect the device characteristics of IGZO TFTs [38]. Unfavorable oxygen deficiencies in IGZO thin films reduce the device stability and electrical performance of IGZO TFT devices. 

Thus, the plasma-treated a-IGZO thin film obtained using an oxygen flow ratio of 33% (sample C), with a low O_II_/(O_I_ + O_II_) XPS intensity ratio of 0.28 was employed as the channel layer for the fabrication of the SG and DG IGZO TFT devices in this study. The use of low-temperature thermal annealing to ensure high performance of IGZO TFT devices is essential for preserving the amorphous structure of the a-IGZO channel layer and reduced donor states as well as the off-current of a-IGZO TFT devices [33,39,40,41].

### 3.2. Hall-Effect Measurements

**Figure 3:**
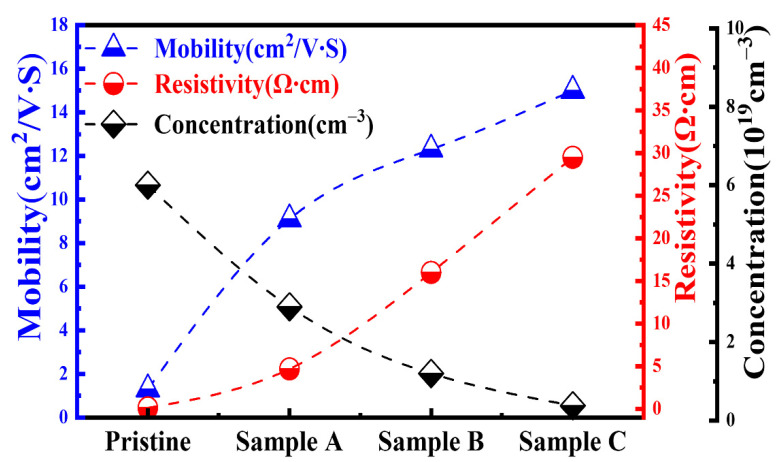
Hall measurement results of the pristine sample and samples A, B, and C.

To investigate the electrical properties of IGZO thin films treated with Ar–O_2_ mixed plasma, Hall measurement was employed to determine the carrier mobility and carrier concentration of the pristine sample and samples A, B, and C. Figure 3 shows the thin-film resistivity (ρ), carrier Hall mobility (μ), and carrier concentration (n) of the pristine sample (without any O_2_ process flow) and samples A, B, and C. This figure shows that the pristine sample had low resistivity (0.13 Ω·cm) and a high carrier concentration of 6 × 10^19^/cm^3^. However, upon increasing the O_2_ gas flow ratio to 33% in the plasma treatment process, the electron concentration of the IGZO thin film decreased markedly from 6 × 10^19^/cm^3^ to 3.8 × 10^18^/cm^3^, and the resistivity increased from 0.13 Ω·cm (pristine sample) to 29.5 Ω·cm (sample C). 

Because the electrons in the conduction band of oxide-based semiconductors originate in the interstice of metal ions and oxygen vacancies, both can act as donors to provide the extra electrons in the oxide semiconductor [42]. Therefore, the increased thin-film resistivity was caused by the reduction of the carrier concentration due to fewer oxygen vacancies in the IGZO thin film upon increasing the oxygen gas flow ratio in the plasma treatment. Additionally, the carrier mobility increased significantly from 1.6 cm^2^/V·s for the pristine sample to 9.1, 12.3, and 15.0 cm^2^/V·s for samples A, B, and C, respectively. The increase in carrier mobility corresponded to an improvement of the thin-film quality caused by the reduction of oxygen vacancies and surface roughness [42,43,44,45], as verified in the AFM investigation discussed shown below.

### 3.3. AFM

**Figure 4:**
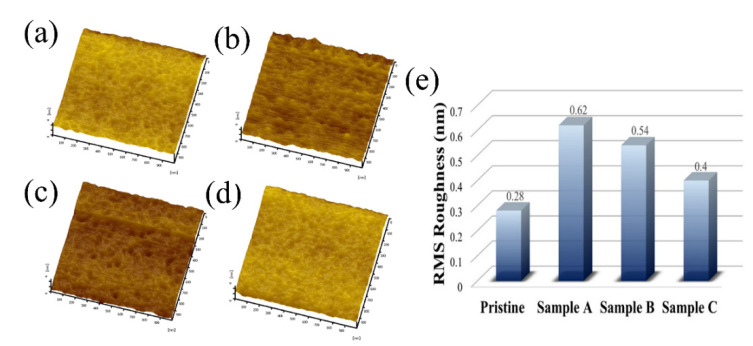
AFM images for the (**a**) pristine sample and samples (**b**) A, (**c**) B, and (**d**) C. (**e**). Surface RMS roughness measured for the pristine sample and samples A, B, and C.

To study the surface roughness of the IGZO thin films after plasma treatment with different O_2_ ratios, AFM was employed for surface morphology measurements for pristine sample and samples A, B, C and D, as shown in Figure 4a–d, respectively. The surface root mean square (RMS) results of the AFM measurements in Figure 4e show that the surface roughness was 0.28 nm for the pristine sample. However, the surface roughness of the IGZO thin films was higher when Ar–O_2_ mixed plasma treatment was applied owing to the physical damage caused by ion bombardment. The increased surface roughness led to higher interfacial trap density and an unexpected leakage current path as well as a poorer *S.S.* and higher I_OFF_ of the IGZO TFT device [46]. However, the thin-film surface roughness was found to decrease from 0.62 to 0.54 and 0.40 nm when the O_2_ gas flow ratio was increased from 16% to 20% and 33% during the Ar–O_2_ gas plasma treatment for samples A, B, and C, respectively. Reducing the thin-film surface roughness was considered to contribute to the improvement in carrier mobility because of a weakened surface scattering effect. Moreover, the low surface roughness in sample C could be useful in reducing the contact resistance between the source and drain electrodes and the IGZO channel layer in the IGZO TFT device.

### 3.4. Device Characteristics

**Figure 5:**
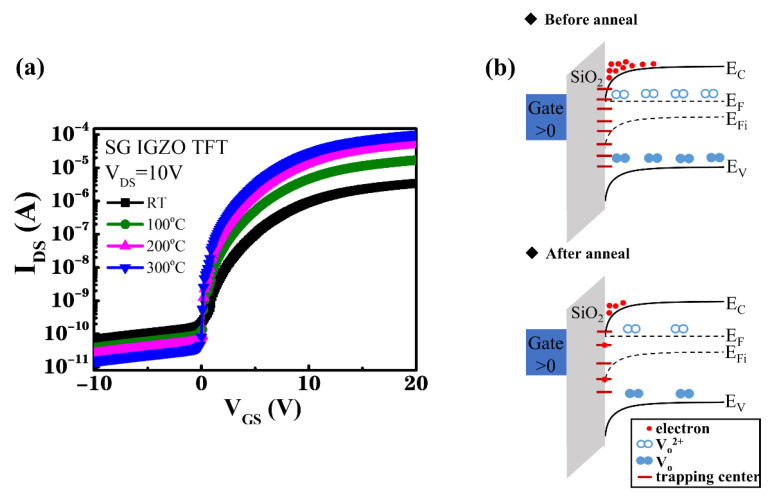
(**a**) I_DS_–V_GS_ transfer characteristics of SG TFTs with a 33% O_2_ plasma–treated IGZO channel layer and annealed at temperatures from RT to 300 °C. (**b**) Energy band diagrams for the SG TFTs before and after the thermal annealing process.

**Figure 6:**
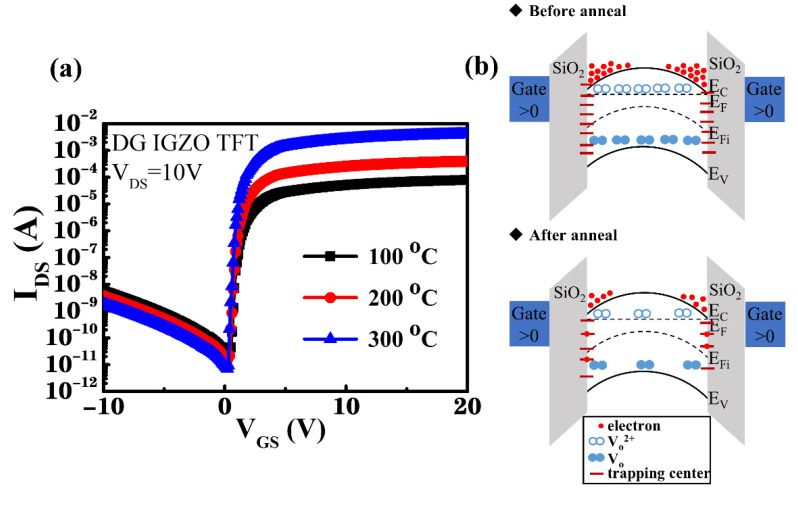
(**a**) I_DS_–V_GS_ transfer characteristics of a DG TFT with a 33% O_2_ plasma–treated IGZO channel layer and annealed at temperatures of 100, 200, and 300 °C. (**b**) Energy band diagrams for the DG TFTs before and after the thermal annealing process.

To improve the characteristics of TFT devices, the oxygen-plasma-treated IGZO channel layers were subjected to thermal annealing at annealing temperatures ranging from room temperature (RT) to 300 °C. The I_DS_–V_GS_ transfer characteristics of the SG and DG IGZO devices annealed at various temperatures were n-type transistor characteristics, as shown in Figure 5a and Figure 6a, while the corresponding illustrations of energy band diagrams for the SG and DG TFTs before and after thermal annealing process were shown in Figure 5b and Figure 6b, respectively. V_DS_ was controlled to 10 V as V_GS_ was increased from −10 to 20 V.

Figure 5a shows that the device characteristics of the SG IGZO TFT device improved upon increasing the annealing temperature from RT to 300 °C; Table 1 summarizes the relevant device performance. The I_ON_/I_OFF_ current switching ratio increased from 7.55 × 10^5^ to 9.93 × 10^6^, and the V_TH_, which was extracted from the linear extrapolation of the square root of I_DS_ versus V_GS_, decreased from 2.2 to 1.1 V upon increasing the annealing temperature from RT to 300 °C. The field-effect mobility μ of the IGZO TFT device was obtained from the drain current in the linear region by using the following equation [38,47]:

(1) $$\mu =\frac{L}{C_{ox}WV_{DS}}\times g_{m}$$

where *μ* is the field-effect mobility; $g_{m}$ is defined as $\left(\partial I_{DS}\right)/\left(\partial V_{GS}\right)$; *C_ox_* values are the unit capacitances of the gate dielectric as 1.38 × 10^−4^ and 2.65 × 10^−4^ F/m^2^ for 250 nm-thick SiO_2_ and Si_3_N_4_ layers (which the dielectric constants are 3.9 and 7.5), respectively. For the DG TFT, the C_ox-dual_ is the combination of C_ox-top_ and C_ox-bottom_. *W* is the channel width, and *L* is the channel length. The calculation shows that the field-effect carrier mobility increased from 18.0 to 38.8 cm^2^/V·s. The *S.S.* and total trap density (*N_t_*) were calculated using Equations (2) and (3), respectively [48]:

(2) $$S.S.=\frac{dV_{GS}}{d\log I_{DS}}$$

(3) $$N_{t}=\left[\frac{S.S.\log(e)}{kT/q}-1\right]\frac{C_{ox}}{q}$$

where *q* is the electron charge; *T* is the absolute temperature, and *k* is the Boltzmann constant. The *S.S.* decreased from 1.25 to 0.7 V/decade with a decrease in *N_t_* from 1.72 × 10^12^ to 9.27 × 10^11^ cm^−2^·eV^−1^ as the annealing temperature was increased from RT to 300 °C, indicating an effective reduction of the number of interfacial trapping centers by conducting thermal annealing.

Because an excess of oxygen atoms was provided by the oxygen plasma treatment, leading to material damages by Ar–O_2_ plasma bombardment, reduction of the oxygen-related defects and interfacial carrier trapping centers by using the thermal annealing process was essential, as schematically shown as the energy band diagrams in Figure 5b and Figure 6b [33]. Additionally, residual stress was released through the annealing process, resulting in a dense IGZO thin film with lower oxygen vacancy density and enhanced device characteristics for the IGZO TFT [49,50,51,52,53,54]. To further improve the characteristics of the IGZO TFT, the DG structure was used to modulate the channel position to prevent the surface scattering effect of the transport carriers. Figure 6a shows the I_DS_–V_GS_ transfer characteristics of the DG TFTs obtained using annealing temperatures of 100–300 °C. Furthermore, Table 2 summarizes the related device characteristics.

The plasma-treated DG IGZO TFT that was thermally annealed at 100 °C exhibited high field-effect carrier mobility of 39.7 cm^2^/V·s with a threshold voltage (V_TH_) of 0.8 V and I_ON_/I_OFF_ current switching ratio of 3.64 × 10^6^. When the annealing temperature was increased to 200 °C and 300 °C, the plasma-treated DG IGZO TFT showed considerably improved field-effect carrier mobility of 42.6 and 58.8 cm^2^/V·s along with increased I_ON_/I_OFF_ current switching ratio of 2.73 × 10^7^ and 5.46 × 10^8^, respectively. Additionally, the *S.S.* decreased from 0.27 to 0.12 V/decade, and *N_t_* decreased from 3.05 × 10^11^ to 8.75 × 10^10^ cm^−2^·eV^−1^. These measurement results show that the annealing process improved the device characteristics of the plasma-treated DG IGZO TFTs, which could have been caused not only because of the reduction in the number of oxygen vacancies but also by hydrogen atom diffusion into the IGZO channel layer. Following the PECVD growth of the thin-film Si_3_N_4_ passivation layer for the fabrication of the DG TFTs, hydrogen atom diffusion was investigated, which contributed to the enhancement of thin-film conductivity and stability by the formation of a stable structure through the annealing process [55].

**Figure 7:**
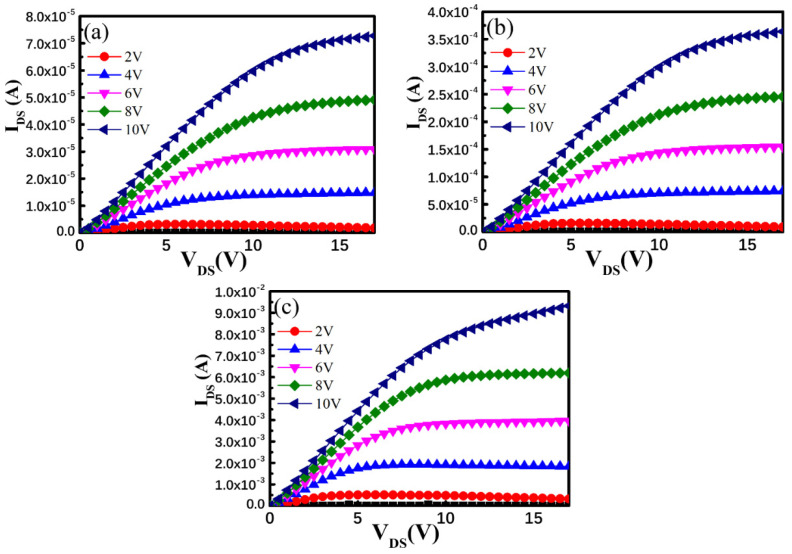
I_DS_–V_DS_ output characteristics of a DG TFT annealed at (**a**) 100 °C, (**b**) 200 °C, and (**c**) 300 °C.

Figure 7a–c shows the output characteristics (I_DS_–V_DS_) of DG TFTs annealed at 100, 200, and 300 °C, respectively. V_DS_ was set with a sweep range of 0–17 V with corresponding V_GS_ of 2,4, 6, 8, and 10 V. All TFT devices exhibited clear current saturation characteristics with a steep current increase in the low V_DS_ range and favorable ohmic characteristics between the channel layer and the source and drain electrodes. Additionally, the saturation drain current I_DS_ of the plasma-treated DG TFTs increased considerably from 7.28 × 10^−5^ A to 9.32 × 10^−3^ A at the bias condition of V_GS_ = 10 V and V_DS_ = 17 V upon increasing the annealing temperature from 100 °C to 300 °C, indicating that the thermal annealing eliminated interfacial carrier trapping centers caused by the Ar–O_2_ plasma ion bombardment of the DG TFTs. Additionally, the DG TFTs subjected to thermal annealing showed considerably improved drain current driving capability and substantially higher saturation drain current compared with those of the SG TFT. When operating the DG TFT device, a positive bias was applied to the top and bottom gate electrodes, causing the accumulation of conduction electrons at the interface at both sides of the IGZO channel layer, which were modulated by the upper and lower gates, respectively. Therefore, the formation of the two components of the accumulated conduction electrons led to an increase in the conductivity of the IGZO channel layer. Consequently, the I_DS_ drain current of the DG TFT devices was much higher than that of the SG TFT device. Furthermore, the *S.S.* of the DG TFT was improved because of the reduced interfacial surface states and the enhanced current drivability resulting from the bulk accumulation as well as a rapid filling of the surface states in a DG design. This design effectively lifted the Fermi level toward the conduction band in a bent energy band alignment at both interfaces of the IGZO layer because both gates were positively biased [35]. Additionally, improved field-effect carrier mobility was observed compared with that of the SG TFT owing to the weaker vertical electrical field between the two positively biased DG electrodes [34]. Therefore, the position of the conductive transporting carrier path was modulated to protect the transporting carriers from the influence of the interfacial carrier scattering effect resulting from the Ar–O_2_ plasma treatment process. This led to improved field-effect carrier mobility in the plasma-treated DG IGZO TFT subjected to thermal annealing.

Because of the absorption of moisture and oxygen from the atmosphere by the IGZO back-channel layer, an increase in the leakage current of the device with the shift in V_TH_ was observed in the IGZO TFT devices. To study the reliability of the operating characteristics of the device, negative gate-bias stress (NBS) and positive gate-bias stress (PBS) measurements were performed on the DG IGZO TFT devices annealed at 100–300 °C in this study.

**Figure 8:**
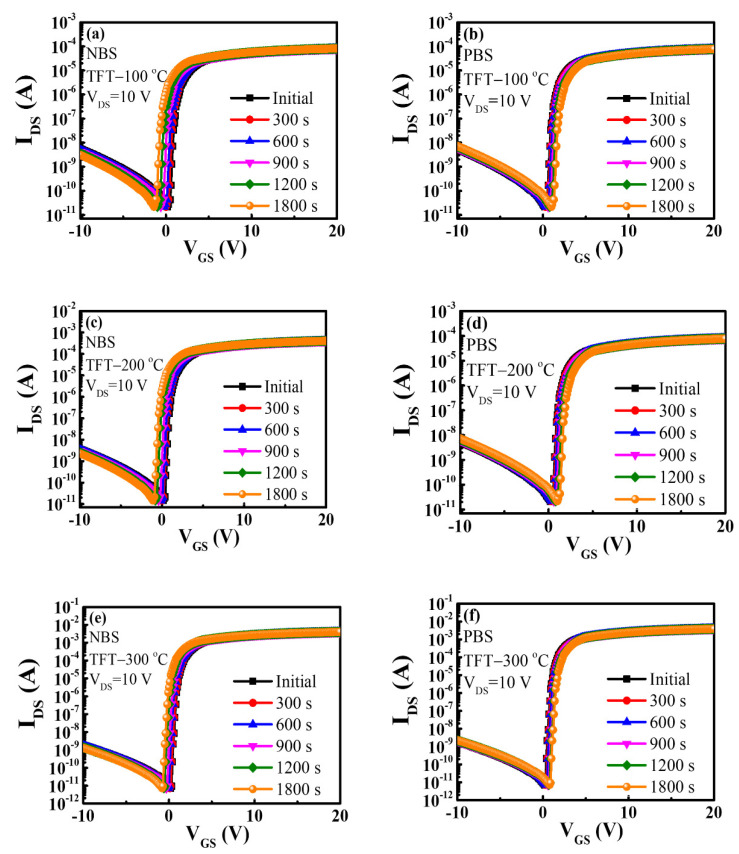
Evolution of transfer characteristics of DG IGZO TFTs with negative bias stress of −10 V for TFTs annealed at (**a**) 100 °C, (**c**) 200 °C, and (**e**) 300 °C and with positive bias stress of 10 V for TFTs annealed at (**b**) 100 °C, (**d**) 200 °C, and (**f**) 300 °C, respectively.

**Figure 9:**
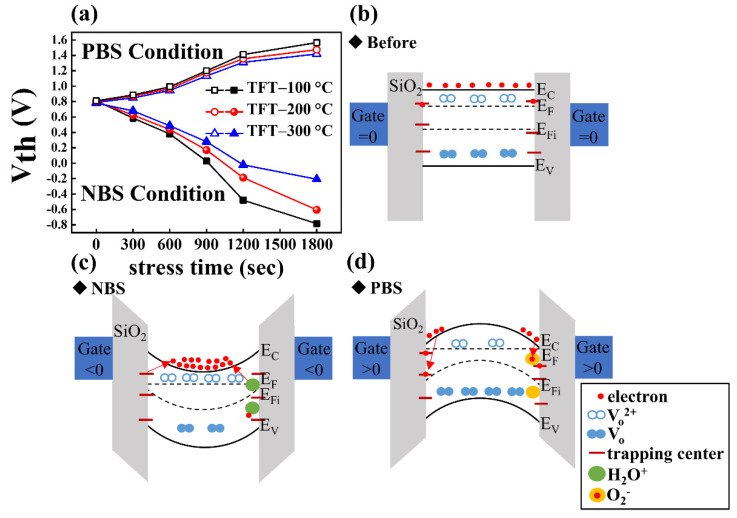
(**a**) The ΔV_TH_ of DG IGZO TFTs with PBS and NBS tests for TFT from the initial condition to the annealed condition at 300 °C. The energy band diagrams for the DG TFTs before and after NBS and PBS tests are shown in (**b**–**d**), respectively.

The transfer curves obtained from NBS and PBS measurements for DG IGZO TFTs annealed at 100, 200, and 300 °C are shown in Figure 8a,b, Figure 8c,d, and Figure 8e,f, respectively, for a fixed V_GS_ bias of ±10 V and varied bias durations of 0, 300, 600, 900, 1200, and 1800 s. The corresponding V_TH_ values for the transfer curves in Figure 8 were extracted and summarized in Figure 9a for clear comparison. The transfer curves were obtained by sweeping V_GS_ from −10 to 20 V while the source electrode was grounded, and the drain voltage was 10 V. The NBS reliability test results for DG IGZO TFTs annealed at 100, 200, and 300 °C are shown in Figure 8a,c,e, respectively. The measurement results indicated that the threshold voltage was shifted in the negative direction with the V_TH_ shift level (ΔV_TH_) by −1.6 V for the TFT device annealed at 100 °C with a stress duration of 0–1800 s. As the annealing temperature was increased to 200 °C and 300 °C, ΔV_TH_ decreased to −1.4 and −1.0 V, respectively; this indicated a reduction of the number of interfacial trapping centers and improvement of the device operation stability with an increase in the annealing temperature. The negative V_TH_ threshold voltage shift of the TFT devices that was observed with increasing stress duration was caused by the trapping centers absorbing moisture, as indicated by the comparison of the energy band diagrams for the unstressed and NBS-tested TFTs in Figure 9b,c; this led to the release of electrons, resulting in more electrons in the IGZO channel layer. Additionally, the ionization of the oxygen vacancies to form Vo^2+^ led to increased electron concentration. Therefore, a negative V_TH_ shift was seen in IGZO TFT devices in NBS reliability measurements [39].

The PBS reliability test results for DG IGZO TFTs annealed at 100, 200, and 300 °C are shown in Figure 8b,d,f, respectively. A positive threshold voltage shift ΔV_TH_ of 0.8 V was discovered for the TFT device annealed at 100 °C for a stress duration of 0–1800 s. When the annealing temperature was increased to 200 °C and 300 °C, ΔV_TH_ decreased to 0.7 and 0.6 V, respectively. The positive V_TH_ shift in the PBS measurement can be explained by the absorption of oxygen molecules by the IGZO back-channel layer, as indicated by the energy band diagram in Figure 9d; this resulted in the formation of oxygen ions with trapped electrons located in the IGZO channel layer. In addition, electrons were trapped by the channel/insulating interfacial electron trapping centers, leading to an increase in V_TH_ [56].

The lower surface roughness and fewer oxygen deficiencies of the plasma-treated IGZO thin film obtained using an oxygen flow ratio of 33% (sample C) caused a reduction in moisture absorption and the number of electron trapping centers at the IGZO back channel layer. This result indicated that Ar–O_2_ plasma–treated IGZO thin films annealed at high temperature contributed not only to the improvement of the electrical characteristics of the device—such as the field-effect carrier mobility, I_ON_/I_OFF_ current ratio, and *S.S.*—but also reduced the *N_t_* defect density and improved the device bias stress stability given the reduction in ΔV_TH_ in the PBS and NBS reliability tests [57,58,59].

## 4. Conclusions

In this study, IGZO channel layers were plasma-treated using an HDP Ar–O_2_ plasma mixture at an oxygen flow ratio of 16–33%. Upon increasing the oxygen flow ratio in the plasma to 33% (sample C), AFM measurements revealed that the surface roughness decreased by 0.40 nm, and XPS revealed a decrease in the oxygen vacancy density in the IGZO thin film. Additionally, the carrier concentration decreased to 3.8 × 10^18^/cm^3^ whereas the carrier Hall mobility increased to 15 cm^2^/V·s.

The SG and DG IGZO TFT devices treated with 33% O_2_ plasma and annealed at 100, 200, and 300 °C exhibited n-type transistor characteristics. For the SG IGZO TFT device, the I_ON_/I_OFF_ current switching ratio, *S.S.*, and field-effect carrier mobility were found to be 9.93 × 10^6^, 0.7 V/decade, and 38.8 cm^2^/V·s, respectively, when performing thermal annealing at 300 °C. Nevertheless, the plasma-treated DG IGZO TFT device processed at 300 °C performed more highly, with an improved I_ON_/I_OFF_ current switching ratio, *S.S.*, and field-effect carrier mobility of 5.46 × 10^8^, 0.12 V/decade, and 58.8 cm^2^/V·s, respectively. This was caused by the weakened surface scattering effect resulting from damage to the surface during plasma treatment and the greater accumulation of conduction electrons caused by modulation of the carrier transportation path in the DG structure. This, in turn, considerably improved the field-effect carrier mobility. The reliability of DG IGZO TFTs annealed at various temperatures was determined using NBS and PBS measurements. NBS and PBS reliability tests revealed improved device operating stability with a reduction in ΔV_TH_ to −1.0 and 0.6 V, respectively, for the TFT annealed at 300 °C. The results of this study showed that the plasma-treated DG IGZO TFT devices annealed at 300 °C exhibited excellent device performance and operational stability, making them highly promising for applications in next-generation displays.

## Figures and Tables

**Table 1:** Electrical characteristics of SG TFTs with a 33% O_2_ plasma–treated IGZO channel layer and annealed at temperatures from RT to 300 °C.

SG TFT (°C)	V_TH_ (V)	Off Current (A)	I_ON_/I_OFF_	*μ* (cm^2^/V·s)	*S.S.* (V/Decade)	*N_t_* (cm^−2^·eV^−1^)
RT	2.2	4.58 × 10^−11^	7.55 × 10^5^	18.0	1.25	1.72 × 10^12^
100	2	2.56 × 10^−11^	8.74 × 10^6^	36.9	1.09	1.49 × 10^12^
200	1.2	1.65 × 10^−11^	3.14 × 10^6^	37.0	0.74	9.85 × 10^11^
300	1.1	1.04 × 10^−11^	9.93 × 10^6^	38.8	0.7	9.27 × 10^11^

**Table 2:** Electrical characteristics of a DG TFT with a 33% O_2_ plasma–treated IGZO channel layer and annealed at temperatures 100, 200, and 300 °C.

DG TFT (°C)	V_TH_ (V)	Off Current (A)	I_ON_/I_OFF_	*μ* (cm^2^/V·s)	*S.S.* (V/Decade)	*N_t_* (cm^−2^·eV^−1^)
100	0.8	2.17 × 10^−11^	3.64 × 10^6^	39.7	0.27	3.05 × 10^11^
200	0.8	1.45 × 10^−11^	2.73 × 10^7^	42.6	0.20	2.03 × 10^11^
300	0.8	7.23 × 10^−12^	5.46 × 10^8^	58.8	0.12	8.75 × 10^10^

## Data Availability

No new data were created or analyzed in this study. Data sharing is not applicable to this article.
